# Loss of hSef promotes metastasis through upregulation of EMT in prostate cancer

**DOI:** 10.1002/ijc.30604

**Published:** 2017-01-30

**Authors:** Satoshi Hori, Karan Wadhwa, Venkat Pisupati, Vincent Zecchini, Antonio Ramos‐Montoya, Anne Y Warren, David E Neal, Vincent J Gnanapragasam

**Affiliations:** ^1^ Academic Urology Group, Department of SurgeryUniversity of CambridgeCambridgeUnited Kingdom; ^2^ Uro‐oncology GroupCancer Research UK Cambridge InstituteCambridgeUnited Kingdom; ^3^ Department of PathologyAddenbrooke's HospitalCambridgeUnited Kingdom

**Keywords:** Sef, negative regulator, metastasis, prostate cancer, EMT

## Abstract

We have previously reported that the negative signaling regulator Similar Expression to FGF (hSef) is downregulated in prostate cancer and its loss is associated with clinical metastasis. Here, we explored the mechanistic basis of this finding. We first confirmed our clinical observation by testing hSef manipulation in an *in vivo* metastasis model. hSef stable expressing cells (PC3M‐hSef) or empty vector controls (PC3M‐EV) were injected subcutaneously into the lateral thoracic walls of NOD‐SCID gamma mice and lungs were harvested at autopsy. In this model, 6/7 PC3M‐EV xenografts had definitive lung micro‐metastasis whilst only 1/6 PC3M‐hSef xenografts exhibited metastasis recapitulating the clinical scenario (*p* = 0.03). Gene expression studies revealed key perturbations in genes involved in cell motility and epithelial to mesenchymal transition (EMT) along with alterations in cognate signaling pathways. These results were validated in an EMT specific PCR array whereby hSef over‐expression and silencing reciprocally altered E‐Cadherin expression (*p* = <0.001) amongst other EMT markers. Immunohistochemistry of excised tumors from the xenografts also confirmed the effect of hSef in suppressing E‐Cadherin expression at the protein level. Phosphokinase arrays further demonstrated a role for hSef in attenuating signaling of not only ERK‐MAPK but also the JNK and p38 pathways as well. Taken together, these data suggest evidence that loss of hSef may be a critical event facilitating tumor dissemination of prostate cancer through alteration of EMT. Detection of downregulated hSef, along with other negative regulators, may therefore be a useful biomarker heralding a transition to a metastatic phenotype and warrants further exploration in this context.

## Abstract 2

**What's new?**

The loss of negative signaling regulators may be implicated in prostate cancer metastasis, but the underlying mechanisms remain unclear. Here, by using a combination of xenograft, gene expression microarray, phosphokinase array and quantitative PCR techniques, the authors provide first evidence that the negative regulator hSef plays a key role in regulating epithelial to mesenchymal transition (EMT) in prostate cancer, which in turn results in changes in the metastatic ability of tumor cells. The results support the notion that the expression levels of hSef and other negative signaling regulators may be key biomarkers for the identification of triggers for metastasis.

Aberrant intracellular signaling is considered one of the hallmarks of cancer.^1^, ^2^ Intracellular signaling however is subject to different levels of regulation that serve to attenuate the eventual impact of stimulation.^3^ Negative signaling regulators (NSR) are feedback‐induced mechanisms that are innate to the cell. There is now emerging evidence that NSR are themselves altered in the transition from benign to malignant cells.^4^ Similar Expression to FGF (Sef) has been particularly well characterized as a tumor suppressor in a diverse range of cancers.^5^

The first of the Sef proteins to be discovered was zebrafish Sef (zSef), which consists of a putative transmembrane domain with a tyrosine phosphorylation site juxtaposed to the receptor (type I transmembrane receptor).^6^ Genomic sequence analysis revealed a 15–20% homology to the intracellular domain of the interleukin‐17 receptor (IL17R),^6^, ^7^ giving it the alternative name of interleukin‐17 receptor D (IL17RD). Sef has since been identified in other vertebrates including chick (cSef), mouse (mSef) and human (hSef).^8^, ^9^, ^10^, ^11^ The mechanism by which Sef regulates receptor tyrosine kinase (RTK) signaling remains contentious and is likely to be cell type specific. Tsang *et al*. in co‐immunoprecipitation experiments have shown that zSef interacts at the level of the FGF receptor.^6^ Kovalenko *et al*. have further shown that overexpression of mSef inhibited phosphorylation of FGFR1 and the adaptor protein FRS2.^9^ Sef interaction at the level of the receptor has also been shown in PC‐12 rat medulla cells.^12^ Consistent with these findings, work in our unit in human prostate cancer has shown that hSef is likely to act at or above the level of Ras.^13,14^ Other groups however have proposed that hSef may function further downstream and potentially act as a spatial regulator of ERK signaling.^15^ Despite the uncertainly on the site of hSef action, it is clear that hSef is an important regulator of diverse signaling pathways.^16^ This key regulatory role has raised an important question on its potential role in cancer development and progression.

A number of NSR have been shown to be altered in cancers and loss of expression has been consistently associated with a more aggressive tumor phenotype.^3^ hSef in particular has been investigated in a number of tumor types including endometrial, ovarian, breast and thyroid and shown to be downregulated in cancer.^5^, ^14^, ^16^ In prostate cancer, our group was the first to demonstrate loss of hSef transcript expression in metastatic clinical tumors.^17^ This observation was further confirmed at the protein level in an expanded study.^13^ In a series of 141 cancers, hSef protein was weak or absent in 46% of biopsies from men with bone metastasis but in only 17% of men without metastasis.^13^ This data raises the possibility that hSef may have an important role as a gatekeeper in the development of disseminated disease. In this study, we explored our clinical observation by modeling the effect of hSef manipulation *in vivo* and *in vitro*. Specifically, our objective was to understand the mechanism by which hSef might facilitate prostate cancer metastasis.

## Material and Methods

### Cell lines and xenograft experiments

PC3 cells were obtained from the American Type Culture Collection and the PC3M cell line (a metastatic derivative of PC3) was a gift from Professor H. Leung (Glasgow). PC3M cells were seeded into 90 mm tissue culture dishes and transfected with pCDNA3.1‐Sef‐Myc (gift from Professor Z Chang, Tsinghua University, China) or pcDNA3.1 (Invitrogen, UK) using Lipofectamine 2000 (Invitrogen, UK) before being placed under G418‐sulphate selection for 14–20 days. Individual colonies were removed by trypsinisation and expanded. Cells were maintained in RPMI‐1640 media (Sigma, UK) containing 10% foetal calf serum, termed full medium (FM). For xenograft studies two million PC3M‐hSef or control cells (suspended in 50 μl of PBS and 50 μl of Matrigel solution) were injected subcutaneously into the lateral thoracic walls of NOD‐SCID gamma mice (Charles River, UK; 10 each with PC3M‐hSef and PC3M‐EV cells). The tumor size at the injection site was measured weekly using calipers and was calculated using the formula: volume = (π/6)/abc or (π/6)/abb (if only 2 diameters are available) and a,b,c are the orthogonal axis of the tumor. At autopsy (typically weeks 4–7), the primary tumor and lungs were harvested. *In vivo* experiments were reviewed and approved by the institutional animal welfare committee and performed according to the UKCCCR guidelines.

### H&E and immunohistochemistry

Harvested tissue was fixed in 10% Neutral Buffered Formalin for 24 hrs and stored in 70% ETOH. H&E staining on the harvested lungs were performed and mounted on slides before image capture and storage. Immunohistochemistry against E‐Cadherin and hSef was performed on the harvested xenograft tissue from the primary injection site using the BondMax Autostainer (Leica, UK). Briefly, the process involved the retrieval of antigen by heat retrieval (100°C) followed by incubation with anti E‐Cadherin antibody (Novocastra, UK) or anti hSef antibody (Sigma, UK) at a dilution of 1:25 or 1:500 at room temperature for 15 min, respectively. The samples were then incubated in a polymer secondary system (Leica) and developed with Diaminobenzidine using copper enhancement. All histopathology sections were scored by an experienced uropathologist (AW). The presence of metastasis was defined as >2 separate sites involved with tumor cells or a single area with >5 tumor cells.

### Illumina microarray, EMT specific PCR array and real‐time PCR

Total RNA was isolated from PC3M‐hSef and PC3M‐EV cells maintained in FM using the RNeasy Mini Kit (Qiagen, UK). Microarray experiments were performed using the HT12v4 beadchip (Illumina, UK). Gene ontology analysis on the resultant data was performed using Ingenuity Pathway Analysis (Qiagen, UK) and MetaCore^TM^ (Thomson Reuters, UK) software with all genes that were differentially expressed by ≥2 fold (up or downregulated) with *p* < 0.01 considered for analysis. Changes in expression of EMT related genes in PC3M‐Sef and PC3M‐EV cells were investigated using the EMT specific RT^2^ Profiler PCR Array (Qiagen, UK). In knockdown experiments PC3 cells were grown in FM for 24 hrs before being transfected with 33 nM of hSef siRNA or Scramble (Scr) control (Dharmacon, UK). ERK‐MAPK inhibition experiments were performed using 30 μM of ERK inhibitor FR180204 (Sigma, UK) suspended in FM. Real‐time PCR analysis were performed using primers obtained from the TaqMan^®^ Gene Expression Assay catalogue (Applied Biosystems, UK) or using in‐house designed primers with SYBR green mastermix (Roche, UK). The sequence of our in‐house primers was as follows: hSef(F) CTGCTCCGTCTTCTTTACGG, hSef(R) GTGATGTTGTACAGCCCACTGTT, E‐Cadherin(F) AGCGTGTGTGACTGTGAAGG, E‐Cadherin(R) CAGCAAGAGCAGCAGAATCA, GAPDH(F) GAAGGTGAAGGTCGGAGTC, GAPDH(R) TGGAAGATGGTGATGGGATT. All experiments were assayed on the Lightcycler^®^ 480 Real‐time PCR machine (Roche, UK) using GAPDH as a housekeeping reference.

### Western blotting and phosphokinase arrays

Cell lysates of hSef knockdown (or Scr control) PC3 cells were obtained at various time‐points post stimulation with FM. Cells were lysed directly using SDS sample buffer containing 10% β‐mercaptoethanol. Samples were denatured and separated using SDS–PAGE. Proteins were transferred onto a PVDF membrane and probed with primary antibodies against total or phosphorylated ERK1 and ERK2 (Cell Signaling Technologies, UK) followed by incubation in the appropriate horseradish peroxidase‐conjugated anti‐rabbit or anti‐goat secondary antibodies (Amersham Life Science). Separate aliquots of these preparations were used in the R&D Systems (UK) Proteome Profiler Phosphokinase antibody array (PKA). Changes in phosphorylation were calculated against pre‐stimulation samples using image quantification as per the manufacturer's instructions.

### Statistical analysis

Data was analysed using the student's *t* test for unpaired samples or Fisher's exact test for categorical data. *p* < 0.05 was considered statistically significant.

## Results

### hSef inhibits tumor dissemination in an *in vivo* metastatic model

**Figure 1:**
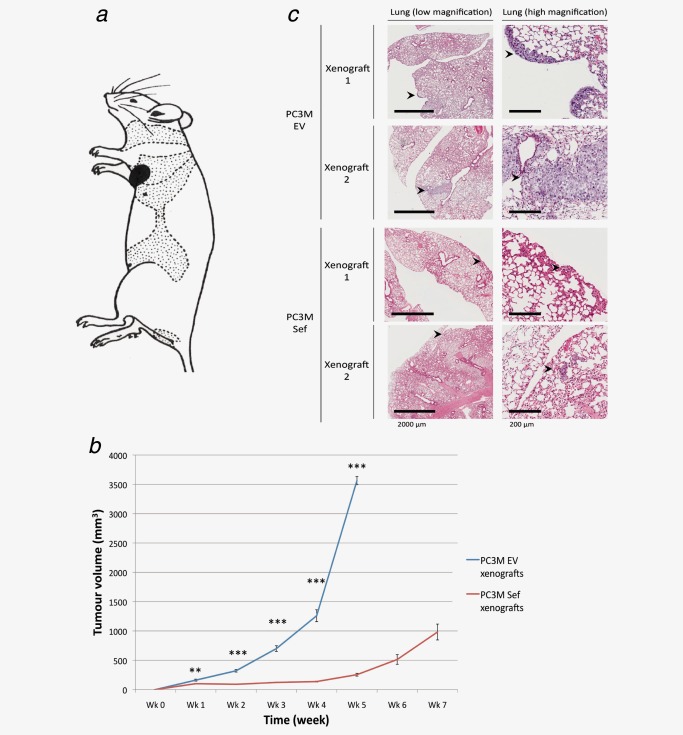
hSef overexpression reduces the metastatic ability of prostate cancer cells *in vivo*. (*a*) Site of hSef overexpressing PC3M cells (PC3M‐hSef) or control (PC3M‐EV) cells injected subcutaneously into the lateral thoracic walls of NOD‐SCID gamma (NSG) mice. (*b*) Serial measurement of tumor volumes at the primary injection site. PC3M‐hSef cells grew slower and formed smaller tumors compared to PC3M‐EV cells. Error bars refer to the range of tumor sizes at each time point and represent 10 animals at each time point (*c*) Representative images of the lung sections of xenografts demonstrating the sites of metastasis with prostate cancer cells. Note that PC3M‐hSef bearing mice produced significantly smaller metastatic deposits compared to their EV counterparts. (**p=<0.01, ***p = <0.001).

Previously, we have demonstrated that hSef expression is closely linked with tumor aggressiveness, with reduced expression being a feature of high‐grade and metastatic clinical prostate cancer.^13^ To corroborate this finding *in vivo,* a xenograft model was adapted from a previously reported protocol for prostate cancer lung metastasis using PC3M.^18^ This cell line is known to have very low levels of endogenous hSef.^17^ PC3M‐EV and PC3M‐Sef cells were used to produce lateral thoracic wall tumors in immune‐deficient mice (Fig. 1 *a*). The generated tumors exhibited significantly different dynamics with PC3M‐Sef tumors growing at a much slower rate compared to PC3M‐EV tumors (Fig. 1 *b*). Harvested lung tissue was assessed for the presence of lung metastasis following autopsy. In total, only two out of ten xenografts injected with PC3M‐hSef had definitive evidence of lung metastasis (Fig. 1 *c*). In contrast, PC3M‐EV readily metastasized to the lungs with nine out of ten xenografts showing evidence of large pulmonary deposits (*p* < 0.01). In PC3M‐hSef bearing mice, not only were there fewer metastatic deposits, but those cells that did metastasize produced very small lesions in comparison to PC3M‐EV (Fig. 1 *c*). We further compared metastatic incidence matched for final tumor volume. In this analysis, 6/7 PC3M‐EV xenografts (mean volume 1.25 cm^3^) had lung micro‐metastasis whilst only 1/6 PC3M‐hSef (mean volume 1.27cm^3^) exhibited metastasis (*p* = 0.03). Collectively, these findings recapitulate the observations from our clinical studies of a role for hSef in influencing tumor metastasis.^13^ This effect appears to be independent of primary tumor size.

### hSef alters the expression of genes involved in EMT

Given our finding that hSef influences the metastatic ability of prostate cancer cells *in vivo*, we next sought to investigate the possible biological mechanisms by which this effect is permeated. For this, RNA extracted from PC3M‐hSef and PC3M‐EV cells following 24 hrs' stimulation with FM was interrogated for comparative gene expression using the Illumina HT12v4 beadchip. Gene network analysis revealed that the top network of genes most influenced by hSef were those involved in cellular movement, cancer, cellular growth and proliferation. Gene ontology analysis was refined further by enriching according to biological functions. This revealed that genes involved in cell adhesion, Extra Cellular Matrix remodeling and Epithelial to Mesenchymal Transition (EMT) appeared to be the most biologically altered by hSef.

**Figure 2:**
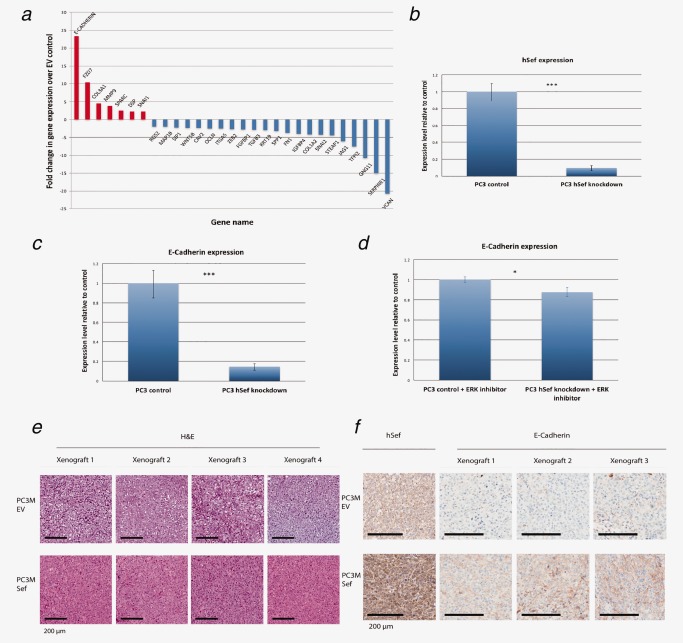
hSef attenuates the expression of EMT genes. (*a*) EMT specific RT^2^ Profiler PCR array revealed that a number of genes involved in the process of EMT were de‐regulated as a result of hSef over‐expression. The gene most upregulated was E‐Cadherin. One of three replicate experiments is shown. (*b*) siRNA against hSef was performed using PC3 prostate cancer cells with a >80% knock‐down efficiency. (*c*) hSef knockdown with FM stimulation resulted in a marked downregulation of E‐Cadherin expression (>80%). Error bars represent the mean of three experiments done in triplicate. (*d*) The experiment was repeated using an ERK‐inhibitor (FR18020) which revealed that ERK signaling blockade significantly reduced the downregulation of E‐Cadherin expression that was seen as a result of hSef knockdown. Error bars represent the mean of the experiment performed in triplicate. (*e*) H&E sections from the primary xenograft tumor injection sites. PC3M‐EV cells were histologically more segregated in comparison with the hSef over‐expressing PC3M cells. (*f*) Immunohistochemistry against hSef protein confirms high expression in PC3M‐Sef but not in PC3M‐EV tumors. E‐Cadherin protein immunohistochemical expression was noted to be absent or very low in PC3M‐EV tumors but increased in hSef over‐expressing xenografts. Representative tumors are shown for both E and F. (*p = <0.05, ***p = <0.001).

We next pursued this observation by investigating the effect of hSef using an EMT gene specific PCR array. In this assay, hSef overexpression with FM stimulation resulted in a significant upregulation of E‐Cadherin expression, a key adhesion molecule involved in EMT (Fig. 2 *a*). In addition, a number of genes involved in upregulating EMT were concomitantly suppressed including SIP1, ZEB2, WNT5B, ITGA5, IGFBP4, STEAP1 and SNAI2 (Fig. 2 *a*). The most markedly downregulated gene was Versican, a chondroitin sulphate proteoglycan known to increase cell migration, growth and metastasis.^19^ We next tested the converse effect of hSef suppression on E‐Cadherin using targeted siRNA in PC3 cells (known to express high levels of endogenous hSef^17^ (Fig. 2 *b*). qPCR analysis revealed that the knock‐down of hSef in PC3 cells stimulated with FM resulted in a significant decrease (>80%) in the expression levels of E‐Cadherin compared to non‐targeting controls (Fig. 2 *c*). Given the central role of hSef in attenuating the ERK‐MAPK pathway, we next investigated whether hSef alters the expression of E‐Cadherin through this pathway. To test this, the above experiment using PC3 control and hSef knockdown cells were repeated using an ERK inhibitor (FR18020). Our results revealed that ERK inhibition significantly reduced the downregulation of E‐Cadherin expression that was seen as a result of hSef knockdown (Fig. 2 *d*). Collectively, our results suggest that hSef influences E‐Cadherin expression through modulation of the ERK‐MAPK pathway.

To corroborate our observation that hSef influences E‐Cadherin expression, histological sections obtained from the primary xenografts tumor sites were subjected to immunohistochemistry for E‐Cadherin protein. On histological features alone PC3M‐hSef tumors appeared more densely packed compared to PC3M‐EV (Fig. 2 *e*). Concomitantly, PC3M‐hSef expressed markedly higher levels of E‐Cadherin compared to EV tumors (Fig. 2 *f*).

### hSef expression simultaneously attenuates ERK, p38 and JNK pathways

**Figure 3:**
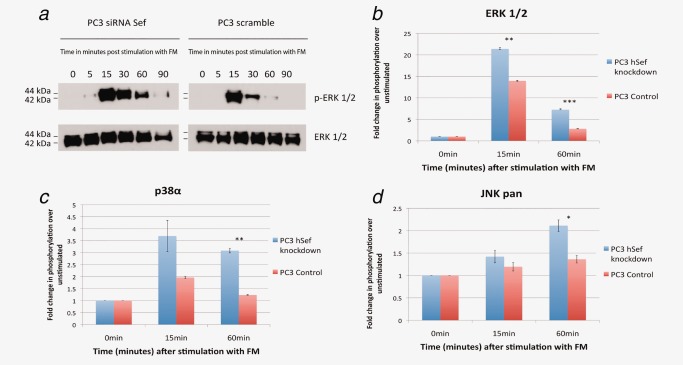
hSef simultaneously attenuates the ERK, p38 and JNK MAPK signaling pathways in prostate cancer cells. (*a*) Western blot on hSef knockdown PC3 cells resulted in an increase in phosphorylation of ERK 1/2 (p‐ERK) in response to growth factor rich media stimulation (FM). One of three replicate experiments is shown. (*b*) hSef knockdown in PC3 cells stimulated with FM also resulted in an increase in the phosphorylation of ERK 1/2 MAPK on the PKA assay, recapitulating the results from the western blot shown in A. (*c*) hSef knockdown also resulted in an increase in phosphorylation of p38 and (*d*) increased phosphorylation of JNK. One representative of 2 repeat phosphokinase array experiments is shown. (*p = <0.05, **p = <0.01, ***p = <0.001).

The role of hSef in modulating the ERK‐MAPK pathway is well described^7^, ^9^, ^12^, ^13^, ^14^, ^15^ and our findings adds further weight to the importance of this pathway in attenuating the expression of E‐Cadherin, a key adhesion protein involved in the EMT process. It is currently not known however, whether hSef has any effect on other intracellular signaling pathways that are involved in mediating EMT such as p38 and JNK.^20^ To investigate this, hSef expression was suppressed using siRNA (or Scr controls) in PC3 cells and stimulated with FM before being tested in a phosphokinase array (PKA). We re‐confirm the robustness of our model system by testing for ERK phosphorylation using western blot. As expected, hSef knockdown resulted in a significant enhancement in both intensity and duration of ERK phosphorylation (Fig. 3 *a*). This was matched in the PKA panel whereby hSef silencing resulted in a significant increase in the phosphorylation of ERK 1/2 compared to scramble transfected cells in response to FM stimulation particularly at the 15 and 60‐min time‐points (Fig. 3 *b*). Across the rest of the panel, hSef silencing also resulted in enhanced phosphorylation of p38 (Fig. 3 *c*) with a similar trend also seen for JNK (Fig. 3 *d*). These results suggest that hSef simultaneously attenuates an array of intracellular signaling pathways, which are known to have a role in mediating EMT.

## Discussion

Metastasis is a critical and devastating step in prostate cancer progression.^21^ The biological mechanisms that lead to prostate cancer metastasis remain poorly understood but a common final pathway is through activation of EMT.^22^ In addition to facilitating tumor dissemination, EMT has also been implicated in resistance to radiotherapy and chemotoxic agents.^23^, ^24^ EMT is therefore an important target when considering novel agents in cancer therapy.

There is good evidence to implicate the loss of NSR in prostate cancer metastasis.^4^ hSef in particular, has been consistently shown to be downregulated in advanced and metastatic tumors.^5^, ^17^ To date however, the functional consequences of this downregulation were thought to be primarily due to changes in cellular growth and proliferation. In this study, we have demonstrated that altering hSef expression has a direct effect in modulating EMT. This is consistent with previous reports of a similar role for other NSR. RKIP1 overexpression, for instance, has been shown to suppress EMT in naso‐pharyngeal cancers.^25^ Members of the Sprouty family are known to repress TGFβ‐induced EMT in the context of cataract development.^26^ Recent work from the same group has since shown a similar role for Spreds 1–3 and Sef in the same context.^27^ Furthermore, He *et al*.^28^ recently revealed that hSef plays a role in the negative regulation of EMT in a β‐catenin dependent manner in breast cancer cells. These data corroborate and support our own findings in this study on the role of hSef in attenuating EMT in prostate cancer.

The exact mechanism by which hSef influences EMT in prostate cancer is currently unknown. Recently, hSef overexpression was found to prevent the downregulation of E‐Cadherin in a normal breast epithelial cell line that was forced to undergo EMT in a TGFβ dependent manner.^28^ As TGFβ induced EMT occurs through canonical and noncanonical pathways,^29^ it is conceivable that hSef modulates EMT by attenuating the various MAPK signaling pathways as was seen in the present study. Specifically, in the context of E‐Cadherin it appears that hSef influences its expression, at least in part, through the attenuation of the ERK‐MAPK pathway. Further studies are required to elucidate the exact mechanism by which hSef influences EMT in prostate cancer.

In conclusion, we have demonstrated here first evidence that hSef has a key role in regulating EMT in prostate cancer, which in turn results in changes in the metastatic ability of tumor cells. This we have now demonstrated *in vitro*, *in vivo* and in clinical studies. These findings support the general notion that hSef and other NSR are important tumor suppressors and a loss of expression leads to a metastatic phenotype. Taken together, this argues strongly for the possibility that the expression levels of hSef and other NSR may be key biomarkers in identifying triggers for metastasis. In addition, while the notion of targeting loss of expression proteins is challenging in terms of therapy, overcoming this paradigm may provide a new class of therapeutic agents, which can inhibit tumor dissemination and therefore alter the natural history of the disease.

## References

[1] Hanahan D , Weinberg RA. The hallmarks of cancer. Cell 2000;100:57–70. 10647931 10.1016/s0092-8674(00)81683-9

[2] Hanahan D , Weinberg RA. Hallmarks of cancer: the next generation. Cell 2011;144:646–74. 21376230 10.1016/j.cell.2011.02.013

[3] Niehrs C , Meinhardt H. Modular feedback. Nature 2002;417:35–6. 11986655 10.1038/417035a

[4] Murphy T , Hori S , Sewell J , et al. Expression and functional role of negative signalling regulators in tumour development and progression. Int J Cancer 2010;127:2491–9. 20607827 10.1002/ijc.25542

[5] Zisman‐Rozen S , Fink D , Ben‐Izhak O , et al. Downregulation of Sef, an inhibitor of receptor tyrosine kinase signaling, is common to a variety of human carcinomas. Oncogene 2007;26:6093–8. 17420726 10.1038/sj.onc.1210424

[6] Tsang M , Friesel R , Kudoh T , et al. Identification of Sef, a novel modulator of FGF signalling. Nat Cell Biol 2002;4:165–9. 11802164 10.1038/ncb749

[7] Furthauer M , Lin W , Ang SL , et al. Sef is a feedback‐induced antagonist of Ras/MAPK‐mediated FGF signalling. Nat Cell Biol 2002;4:170–4. 11802165 10.1038/ncb750

[8] Harduf H , Halperin E , Reshef R , et al. Sef is synexpressed with FGFs during chick embryogenesis and its expression is differentially regulated by FGFs in the developing limb. Dev Dyn 2005;233:301–12. 15844098 10.1002/dvdy.20364

[9] Kovalenko D , Yang X , Nadeau RJ , et al. Sef inhibits fibroblast growth factor signaling by inhibiting FGFR1 tyrosine phosphorylation and subsequent ERK activation. J Biol Chem 2003;278:14087–91. 12604616 10.1074/jbc.C200606200

[10] Lin W , Furthauer M , Thisse B , et al. Cloning of the mouse Sef gene and comparative analysis of its expression with Fgf8 and Spry2 during embryogenesis. Mech Dev 2002;113:163–8. 11960706 10.1016/s0925-4773(02)00018-7

[11] Yang RB , Ng CK , Wasserman SM , et al. A novel interleukin‐17 receptor‐like protein identified in human umbilical vein endothelial cells antagonizes basic fibroblast growth factor‐induced signaling. J Biol Chem 2003;278:33232–8. 12807873 10.1074/jbc.M305022200

[12] Xiong S , Zhao Q , Rong Z , et al. hSef inhibits PC‐12 cell differentiation by interfering with Ras‐mitogen‐activated protein kinase MAPK signaling. J Biol Chem 2003;278:50273–82. 12958313 10.1074/jbc.M306936200

[13] Darby S , Murphy T , Thomas H , et al. Similar expression to FGF (Sef) inhibits fibroblast growth factor‐induced tumourigenic behaviour in prostate cancer cells and is downregulated in aggressive clinical disease. Br J Cancer 2009;101:1891–9. 19888221 10.1038/sj.bjc.6605379 PMC2788253

[14] Zhang H , Zhao X , Yan L , et al. Similar expression to FGF (Sef) reduces endometrial adenocarcinoma cells proliferation via inhibiting fibroblast growth factor 2‐mediated MAPK/ERK signaling pathway. Gynecol Oncol 2011;122:669–74. 21663947 10.1016/j.ygyno.2011.05.019

[15] Torii S , Kusakabe M , Yamamoto T , et al. Sef is a spatial regulator for Ras/MAP kinase signaling. Dev Cell 2004;7:33–44. 15239952 10.1016/j.devcel.2004.05.019

[16] Ron D , Fuchs Y , Chorev DS. Know thy Sef: a novel class of feedback antagonists of receptor tyrosine kinase signaling. Int J Biochem Cell Biol 2008;40:2040–52. 18450498 10.1016/j.biocel.2008.03.013

[17] Darby S , Sahadevan K , Khan MM , et al. Loss of Sef (similar expression to FGF) expression is associated with high grade and metastatic prostate cancer. Oncogene 2006;25:4122–7. 16474841 10.1038/sj.onc.1209428

[18] Kozlowski JM , Fidler IJ , Campbell D , et al. Metastatic behavior of human tumor cell lines grown in the nude mouse. Cancer Res 1984;44:3522–9. 6744277

[19] Zheng PS , Wen J , Ang LC , et al. Versican/PG‐M G3 domain promotes tumor growth and angiogenesis. Faseb J 2004;18:754–6. 14766798 10.1096/fj.03-0545fje

[20] Lamouille S , Xu J , Derynck R. Molecular mechanisms of epithelial‐mesenchymal transition. Nat Rev Mol Cell Biol 2014;15:178–96. 24556840 10.1038/nrm3758 PMC4240281

[21] Hori S , Jabbar T , Kachroo N , et al. Outcomes and predictive factors for biochemical relapse following primary androgen deprivation therapy in men with bone scan negative prostate cancer. J Cancer Res Clin Oncol 2011;137:235–41. 20390426 10.1007/s00432-010-0877-9 PMC11828148

[22] Creighton CJ , Gibbons DL , Kurie JM. The role of epithelial‐mesenchymal transition programming in invasion and metastasis: a clinical perspective. Cancer Manag Res 2013;5:187–95. 23986650 10.2147/CMAR.S35171 PMC3754282

[23] Nantajit D , Lin D , Li JJ. The network of epithelial‐mesenchymal transition: potential new targets for tumor resistance. J Cancer Res Clin Oncol 2015;141:1697–713. 25270087 10.1007/s00432-014-1840-y PMC4382462

[24] Mitra A , Mishra L , Li S. EMT, CTCs and CSCs in tumor relapse and drug‐resistance. Oncotarget 2015;6:10697–711. 25986923 10.18632/oncotarget.4037 PMC4484413

[25] He QY , Yi HM , Yi H , et al. Reduction of RKIP expression promotes nasopharyngeal carcinoma invasion and metastasis by activating Stat3 signaling. Oncotarget 2015;6:16422–36. 25915430 10.18632/oncotarget.3847 PMC4599279

[26] Shin EH , Basson MA , Robinson ML , et al. Sprouty is a negative regulator of transforming growth factor beta‐induced epithelial‐to‐mesenchymal transition and cataract. Mol Med 2012;18:861–73. 22517312 10.2119/molmed.2012.00111 PMC3409273

[27] Zhao G , Wojciechowski MC , Jee S , et al. Negative regulation of TGFbeta‐induced lens epithelial to mesenchymal transition (EMT) by RTK antagonists. Exp Eye Res 2015;132:9–16. 25576668 10.1016/j.exer.2015.01.001

[28] He Q , Gong Y , Gower L , et al. Sef Regulates Epithelial‐Mesenchymal Transition in Breast Cancer Cells. J Cell Biochem 2016;117:2346–56. 26950413 10.1002/jcb.25532 PMC5382796

[29] Zhang YE , Non‐Smad pathways in TGF‐beta signaling. Cell Res 2009;19:128–139. 19114990 10.1038/cr.2008.328 PMC2635127

